# Predicting coral dynamics through climate change

**DOI:** 10.1038/s41598-018-36169-7

**Published:** 2018-12-20

**Authors:** Robert van Woesik, Semen Köksal, Arzu Ünal, Chris W. Cacciapaglia, Carly J. Randall

**Affiliations:** 1 Institute for Global Ecology, Florida Institute of Technology 150 West University Boulevard, Melbourne, Florida 32901 United States of America; 2 Department of Mathematical Sciences, Florida Institute of Technology 150 West University Boulevard, Melbourne, Florida 32901 United States of America; 3 Department of Mathematics, Ankara University, Tandogan, Ankara, 06100 Turkey; 4 Australian Institute of Marine Science, PMB 3, Townsville, Queensland 4810 Australia

## Abstract

Thermal-stress events are changing the composition of many coral reefs worldwide. Yet, determining the rates of coral recovery and their long-term responses to increasing sea-surface temperatures is challenging. To do so, we first estimated coral recovery rates following past disturbances on reefs in southern Japan and Western Australia. Recovery rates varied between regions, with the reefs in southern Japan showing more rapid recovery rates (intrinsic rate of increase, *r* = 0.38 year^−1^) than reefs in Western Australia (*r* = 0.17 year^−1^). Second, we input these recovery rates into a novel, nonlinear hybrid-stochastic-dynamical system to predict the responses of Indo-Pacific coral populations to complex inter-annual temperature cycles into the year 2100. The coral recovery rates were overlaid on background increases in global sea-surface temperatures, under three different climate-change scenarios. The models predicted rapid recovery at both localities with the infrequent and low-magnitude temperature anomalies expected under a conservative climate-change scenario, Representative Concentration Pathway (RCP) 4.5. With moderate increases in ocean temperatures (RCP 6.0) the coral populations showed a bimodal response, with model runs showing either recovery or collapse. Under a business-as-usual climate-change scenario (RCP 8.5), with frequent and intense temperature anomalies, coral recovery was unlikely.

## Introduction

The rapid rate of contemporary climate change is seriously affecting terrestrial and marine populations^1,2^, particularly on tropical coral reefs where corals have been living close to their upper thermal limits for millennia^3–5^. Although corals on tropical reefs are adapted to warm waters, they rarely experience an annual temperature range greater than a few degrees Celsius. Warmer than average temperatures combined with high seasonal irradiance can cause a dysfunction in the coral-dinoflagellate symbiosis that leads to coral bleaching, and under extreme conditions leads to mortality^6–10^. There is, however, considerable variability in the range of thermal tolerances among the eight hundred or more extant coral species^11–13^, and there are substantial differences in the rates of population and community recovery from thermal disturbances^14,15^.

The rate of recovery from a disturbance is a function of a system’s stability and resilience^16,17^. Stable, resilient systems recover rapidly; whereas unstable, non-resilient systems recover slowly, or even collapse after disturbances^18^. Moreover, the slowing of recovery usually indicates that a system is deteriorating and may be approaching a critical threshold, beyond which the system switches to an alternate and often undesirable state^19,20^. Indeed, understanding the rates of change and the resilience of systems has become central to our understanding of modern ecology^21,22^.

On modern coral reefs, we frequently ask the question: how quickly will a reef recover from a given disturbance? It is challenging to answer this question, particularly given the complexity of coral reefs and the multitude of nuances that influence recovery^23,24^. Recovery depends on many interacting factors, including species composition and environmental and geographic vicissitudes. Understanding these factors and accurately predicting the recovery rates of coral populations is critical in a rapidly changing climate^25^, which is characterized by ocean warming and an increase in the frequency of acute thermal-stress events^5^. There is, therefore, a need for models that accurately predict the dynamics of coral populations and determine the likelihood of recovery under future climate-change scenarios RCP 4.5, 6.0, and 8.5^26^.

Many models explore the dynamics of coral populations^25–28^, although most models are built on the traditional foundation of Leslie and Lefkovitch matrices^29–31^. The matrix approach is convenient, but does not capture the instantaneous dynamics of a population. A system of differential equations captures the dynamics of change^32,33^, but rarely includes environmental stochasticity and ecological uncertainty. Nonlinear stochastic models, however, do allow for nuances involving uncertain return-periods of thermal events. Here we constructed a nonlinear hybrid-stochastic-dynamical system to model the responses of coral populations to the complexities of sea-surface temperature cycles, which vary stochastically in both frequency and intensity. We estimated recovery rates from disturbances to the coral reefs in southern Japan and Western Australia, which have similar coral-species composition. We then input these recovery rates into a generic model that predicted disturbance and recovery of Indo-Pacific coral populations into the year 2100, superimposed on increasing global sea-surface temperatures.

## Methods

**Figure 1:**
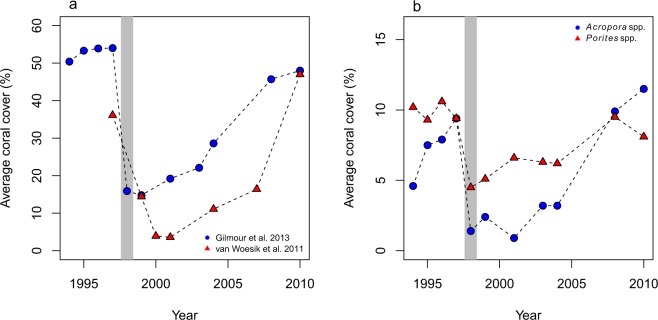
Coral-population data for the Scott Reef system in north Western Australia (Gilmour *et al*.^15^), and for Sesoko Island, southern Japan (van Woesik *et al*.^14^). (**a**) Average coral cover (%). (**b**) Average coral cover (%) of *Acropora* spp. and *Porites* spp. from Gilmour *et al*.^15^. Gray bars indicate the timing of the El Niño-driven mass-bleaching and mortality event between 1997 and 1998. Note that the y-axes differ between panels.

Following a disturbance on a coral reef, and in the absence of further extreme environmental conditions, the change in average precentage coral cover (*P*) through time (Fig. 1) often can be approximated by the logistic growth equation. To add environmental complexity and stochastic extremes we considered that the physiological response of corals depends on temperature stress, which in turn depends on irradiance^32^. A newly constructed hybrid stochastic model, given below, governs the dynamics of that response and the occurrences of extreme-temperature events:

1a $$\frac{dP}{dt}=rP(1-\frac{P}{K})-\gamma TP\,{\rm{for}}\,t\notin ({t}_{k},\,{t}_{k}+h)\,{\rm{and}}\,{t}_{0}\le t\le {t}_{N}{\rm{with}}\,P({t}_{0})={P}_{0},$$

1b $$P(t)=P({t}_{k}){e}^{f(\varepsilon )t},\,{t}_{k}\le t\le {t}_{k}+h\,{\rm{for}}\,k=1,2,\ldots ,\,n\le N,$$

where *r* is the intrinsic rate of increase in coral cover per year, *K* is the steady-state coral cover equilibrium point (%), *T* is the sea-surface temperature (°C), and where *t*_*k*_’s are the years when extreme thermal-stress events occur. *N* is an integer that indicates the number of years we wish to run the simulations, and in our case it was through to the year 2100. In addition, *h* is the duration of a thermal-stress anomaly (weeks), which was extracted from a truncated gamma distribution with a minimum of 2 weeks and a maximum of 8 weeks, and γ is a coefficient that impacts coral populations through temperature changes, which affects percentage coral cover. During an extreme-temperature event, coral cover is modeled by Equation 1b, where *ε*, which we call an ‘extreme-event coefficient’, is the sudden increase in temperature, herein governed by short-term climate oscillations such as El Niño events, which result in mass coral bleaching and mortality. The function *f*(*ε*) controls the dynamics, specific to each coral species or coral assemblage, depending on sudden temperature increases (measured by *ε*). Since the coral populations decrease during temperature anomalies, *f*(*ε*) is a negative function for all times. Using the present biological data the function was estimated as −*ε*^3^. These values would most likely vary geographically and for different coral assemblages, and could vary into the future, with anomalous temperature events not necessarily coupled with El Niño cycles^5^, although the generic construct of the model will remain the same.

Sea-surface temperature (in Celsius), *T*(*t*), was obtained by curve fitting satellite data (see below), using the following equation:

2 $$T(t)={I}_{ave}({a}_{1}\,\cos (t)+{a}_{2}\,\sin (t))+\lambda t+{a}_{3},$$

where *I*_*ave*_ is the annual average of irradiance (in photosynthetic available radiation, PAR), and the parameters *a*_1_ and *a*_2_ are the rates at which irradiance affects the change in temperature (or the rate of change in Celsius per PAR) (in Celsius/PAR). We assume that seasonal irradiance does not vary among years. Here, *λ* is defined as the ‘climate-change coefficient’ (Celsius time^−1^), where *λt* controls the temperature increase over time, in years, and *a*_3_ is the annual average of sea surface temperature (in Celsius) for *λ* = 0 under normal, non-anomalous years (Table 1). To estimate these parameters we used nonlinear optimization using the program Mathematica®.

**Table 1:** Estimated coefficients of sea-surface temperature functions in Equation 2 for each Representative Concentration Pathway (RCP) scenario (IPCC 2013).

Scenarios	*a*_1_	*a*_2_	*a*_3_	*λ*
Modern	−0.003	0.001	25.49	0
RCP 4.5	−0.0001	0.00013	−4.81	0.015
RCP 6.0	0.00002	−0.00005	−16.06	0.021
RCP 8.5	−0.00006	0.00002	−52.08	0.039

The parameters *a*_1_ and *a*_2_ are the rates at which irradiance affects the change in sea-surface temperature, *λ* is defined as the ‘climate-change coefficient’, where *λt* controls the sea-surface temperature increase over time, in years, and *a*_3_ is the annual average of sea-surface temperature for *λ* = 0 under normal, non-anomalous years.

### Biological data

To estimate the parameters in Equation 1, we first estimated recovery rate (*r*) and carrying capacity (*K*). We obtained data sets of coral cover from two geographic regions: Sesoko Island, Okinawa, in southern Japan^11,14^, and Scott Reef in Western Australia^15^. Total coral cover estimates were obtained from Sesoko Island from 1997–2010^11,14^. The corals at Sesoko Island experienced an anomalous thermal-stress event in 1998, which was driven by the El Niño Southern Oscillation, which reduced coral populations significantly^11^. The Sesoko Island reefs also experienced a milder thermal-stress event in 2001 (Fig. 1). In Western Australia, Gilmour *et al*., see ref.^15^, collected data on total cover, and the cover of dominant reef-building genera, including *Acropora* and *Porites*, from Scott Reef from 1994–2010 (Fig. 1). The reefs also suffered coral losses during the 1998 anomalous thermal-stress event (Fig. 1).

We could not, however, with any degree of certainty, simultaneously fit all the unknown parameters in Equation 1a and also predict coral cover into the future. Therefore, we used a two-step process. First, we estimated *r*, *K* and γ as inverse problems using a Bayesian platform. The values from the Bayesian posterior distributions were then fitted to parametric distributions. Second, we used these best-fit distributions to predict the response of coral populations to likely thermal-stress scenarios (see below) through to the year 2100. We estimated *r* and *K* values (Equation 1a) at each study locality as an inverse problem using the logistic growth function within a Bayesian framework using a lag-1 temporal auto-regressive error structure on the residuals (after ref.^34^). The successive *r*-values for the different localities were derived from a sample size of 50,000 from the posterior distribution, estimated using OpenBUGS. These *r-*values, from the posterior distribution, were low, <0.4, and were therefore fitted to Beta distributions using the R package ‘*fitdistrplus*’^35^. Random samples were taken from these estimated Beta distributions for every time step in the predictive model. We used a similar process to estimate γ-values using Equation 3 below. Although temperatures change through the seasons, in the tropics they only range about 3 °C, which we assumed was small enough to fix T, and thereby use mean temperature and the solution of Equation 1a (i.e., Equation 3) to estimate γ-values. Therefore, to derive a meaningful estimate of γ, we treated temperature,*T*, and *K* as constants, and treated *r* as a stochastic parameter, using the following:

3 $$P(t)=\frac{(r-\gamma T){P}_{o}}{\frac{r}{K}\,{P}_{o}(1-ex{p}^{-(r-\gamma T)t})+(r-\gamma T)\,ex{p}^{-(r-\gamma T)t}}.$$

### Temperatures, Thermal stress, El Niño, and Climate Change

Sea-surface temperature (SST) data were obtained for each reef from the Moderate-resolution Imaging Spectroradiometer (MODIS) on board the Aqua satellite platform that was launched in 2002^36^. Average monthly MODIS Aqua Global Level 3 mapped mid-infrared nighttime SSTs from January 2003 (the earliest available date) through December 2010 were obtained at a 4.6 km spatial resolution for each locality (Sesoko Island 26.65854°N 127.8611°E; Scott Reef 14.167959°S, 121.87968°E). Monthly SST records were extracted from each study site and mean monthly SST temperature was calculated.

**Figure 2:**
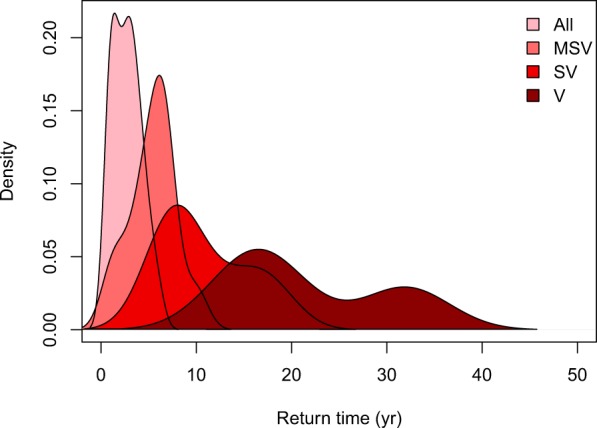
History of the frequency of El Niño events from 1950–2016, where ‘All’ indicates all events, ‘MSV’ indicates moderate, strong, and very strong, ‘SV’ indicates strong and very strong, and ‘V’ indicates very strong events.

To estimate the distribution of the frequency of thermal-stress events, we obtained Oceanic Niño Index (ONI) data from 1950–2016. The ONI is the standard metric used to identify El Niño (warm) and La Niña (cool) events in the tropical Pacific, and is a running 3-month average sea-surface temperature anomaly for the Niño 3.4 region^37^ (5°N–5°S and 120°W–170°W), based on the Extended Reconstructed Sea Surface Temperature (ERSST v4) dataset from the National Oceanic and Atmospheric Administration’s National Climate Data Center. These data were derived from the International Comprehensive Ocean-Atmosphere Dataset at a 2° by 2° spatial resolution (https://www.ncdc.noaa.gov). El Niño events were defined as five consecutive three-month periods, at or above the 0.5 °C anomaly. We categorized El Niño events into one of four strengths based on the magnitude of the anomaly: (1) weak events were characterized as 0.5–0.9 °C anomalies, (2) moderate events were defined as 1.0–1.4 °C anomalies, (3) strong events were defined as 1.5–1.9 °C anomalies, and (4) very strong events were greater than or equal to 2.0 °C anomalies (Table 2). The frequency of thermal-stress events was then estimated for each of four categories: (1) all events; (2) only moderate, strong, and very strong events; (3) only strong and very strong events; and (4) only very strong events (Fig. 2).

**Table 2:** Average and median periodicity of El Niño events from 1950–2016, based on data from the Oceanic Niño Index (https://www.ncdc.noaa.gov).

El Niño-event Periodicities	SST anomaly	Mean ± SD	Median
1. All events	≥0.5 °C	2.7 ± 1.5	3
2. Only moderate, strong and very strong events	≥1 °C	5.4 ± 2.4	6
3. Only strong and very strong events	≥1.5 °C	10.8 ± 4.6	9
4. Only very strong events	≥2 °C	21.7 ± 9.0	18

The periodicities of all events in each of four categories, defined by the strength of the anomalies, were estimated. SD = standard deviation; SST = sea-surface temperature (°C). The strongest events (≥2 °C) were excluded from this study because the infrequent return period lowers the confidence in these events for our relatively short timeframe (to the year 2100).

To estimate the distribution of the frequency of thermal-stress events of each category of El Niño, the number of years in between the start of each event was calculated. The mean, median, and standard deviation of the period in between the anomalous-thermal events were also calculated (Table 2). The frequency of extreme events in the model simulations was drawn from a Poisson distribution where the rate parameter was estimated from the mean of the frequency of thermal-stress events of categories 1–3 (i.e., between 3 and 9 years). The historical record shows that very strong thermal-stress events occurred every 21.7 ± 9 years (median = 18) (Fig. 2); therefore very strong events were excluded from this study because the infrequent return period lowers the confidence in these events for our relatively short timeframe (to the year 2100).

It is expected that, by 2100, sea-surface temperatures will increase by approximately 1.8 °C, 2.2 °C and 3.7 °C for the Representative Concentration Pathways (RCPs) 4.5, 6 and 8.5 respectively^38^. Therefore, the maximum temperature anomaly was set to 3.7 °C bove the mean background temperatures in the models^38^. Based on the RCP predictions, the corresponding climate-change coefficient values, *λ*, in Equation 2 were re-calculated using curve fitting (Table 1).

### Model implementation

The intervals between thermal-stress events were extracted randomly from a Poisson distribution, with values selected between 3- and 9-year intervals (Table 2), and the duration of each thermal-stress event was randomly selected from a gamma distribution lasting anywhere from 2 to 8 weeks over any given summer period. Because seasonal irradiance is unlikely to vary among years^39^, I_ave_ was set at 45 in Equation 2 for all iterations. There was a minimal effect of the λ parameter (in Equation 2) on temperature increases between the years 2000 and 2010, therefore λ (Equation 2) was set to zero for the initial iteration (Table 1). The algorithm used for the model is available in Appendix 1; all the R code and the OpenBugs code is available in the supplementary document.

## Results

**Figure 3:**
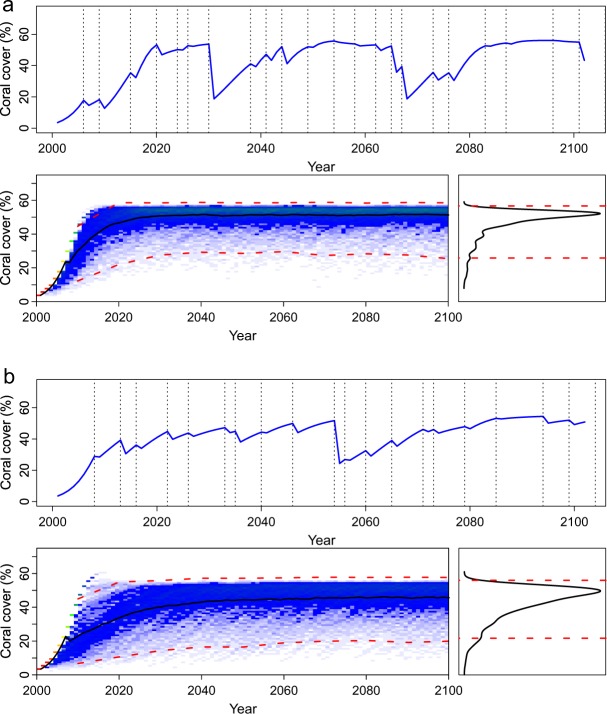
Predictions of % coral cover for (**a**) southern Japan and (**b**) Western Australia through to the year 2100, for sea-surface temperatures predicted under climate-change scenario RCP 4.5 Wm^−2^ (i.e., 1.8 °C) and intermittent temperature anomalies (lambda = 0.0001, gamma = 0.001, *f*(*ε*) is estimated as −*ε*^3^). In both 3a and 3b, the top graph shows one model run through to the year 2100, whereas the bottom left graph shows the results of 1000 model runs as a heat map, and the bottom right graph shows the resulting percentage coral cover estimates in 2100 as a frequency distribution. Vertical black dashed vertical lines indicate thermal-stress events, and the red dashed horizontal lines are credible intervals on the model output distributions.

**Figure 4:**
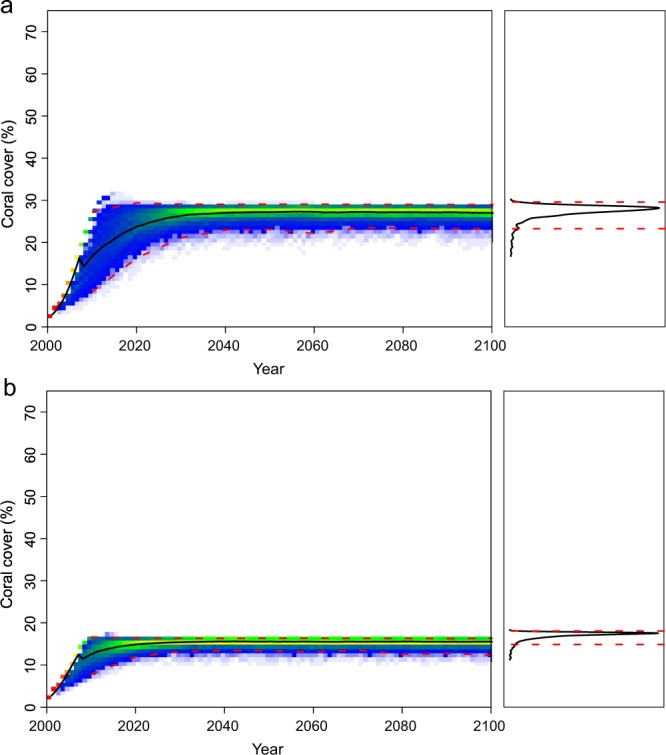
Predictions of % coral cover for (**a**) *Acropora* spp. and (**b**) *Porites* spp. in Western Australia, through to the year 2100, for sea-surface temperatures predicted under climate-change scenario RCP 4.5 Wm^−2^ (i.e., 1.8 °C) and intermittent temperature anomalies (lambda = 0.0001, gamma = 0.001, *f*(*ε*) is estimated as −*ε*^3^). The left graph shows the results of 1000 model runs as a heat map, the right graph shows the resulting percentage coral cover estimates in 2100 as a frequency distribution, and the red dashed horizontal lines are 95% credible intervals on the model output distributions.

The estimated carrying capacities (*K*) were similar between reefs, at ~53% in southern Japan and 64% in Western Australia, although uncertainty, that is the 95% credible intervals, was greater for the reefs in southern Japan than for the reefs in Western Australia (Table 3). The recovery rates, or the intrinsic rates of increase, of the coral populations were higher in southern Japan (*r* = 0.39) than in Western Australia (*r* = 0.17), again with considerably greater uncertainty for southern Japan than for Western Australia (Table 3). The recovery rates of *Acropora* (*r* = 0.19) were higher than the recovery rates of *Porites* (*r* = 0.09) in Western Australia, with similar uncertainty intervals for each genus (Table 4). Under modern temperature conditions with intermittent temperature anomalies, the model predicted that both geographic localities maintained relatively high coral cover, near their carrying capacities (Fig. 3). The predictions for *Acropora* and *Porites* varied, with *Acropora* populations showing greater fluctuations through time than *Porites* (Fig. 4) in Western Australia.

**Table 3:** Estimates of key parameters in Equation 1 from Markov Chain Monte Carlo and Gibbs sampling, using total coral cover from two localities: Scott Reef, Western Australia^15^, and Sesoko Island, southern Japan^14^. All estimates were implemented using uninformative priors.

Total Coral Cover	Parameters	Mean	95% equal-tailed credible intervals
Southern Japan	*r*	0.39	0.055; 0.68
	*K*	52.63	14.93; 74.07
Western Australia	*r*	0.17	0.14; 0.26
	*K*	63.85	42.55; 74.61

The Beta distributions of the *r* -values were *α* = 3.16 and *β* = 5.14 for southern Japan, *α* = 27.01 and *β* = 127.78 for Western Australia, and where *K* is the carrying capacity.

**Table 4:** Estimates of key parameters in Equation 2 from Markov Chain Monte Carlo and Gibbs sampling using *Acropora* spp. coral and *Porites* spp. coral field data from Western Australia^15^. All estimates were implemented using uninformative priors.

Total Coral Cover Western Australia	Parameters	Mean	95% equal-tailed credible intervals
*Acropora* spp.	*r*	0.19	0.16; 0.27
	*K*	39.52	12.34; 72.43
*Porites* spp.	*r*	0.09	0.05; 0.16
	*K*	25.95	10.19; 69.95

The Beta distribution for *Acropora* spp. recovery in Western Australia was estimated at *α* = 35.29 and *β* = 147.51, and for *Porites* spp. was estimated at *α* = 8.33 and *β* = 84.67, and where *K* is the carrying capacity.

**Figure 5:**
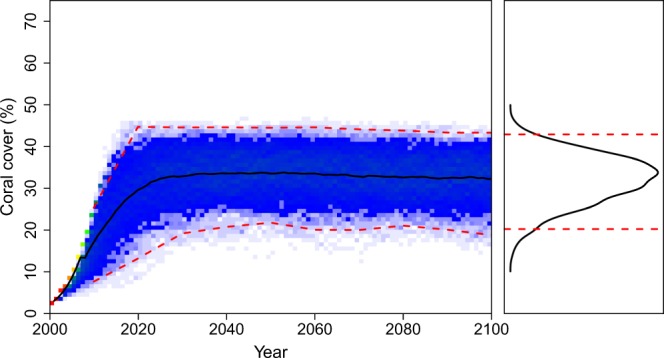
Predictions of % coral cover in Sesoko Island, southern Japan, with *high lambda* = *0*.*0*3*9* (Equation 2) for sea-surface temperatures predicted under climate-change scenario RCP 8.5 Wm^−2^ (i.e., 3.7 °C) and *high gamma* = *0*.*005* (Equation 1a) and *low epsilon* = *3* (i.e., with low intensity of thermal anomalies). The left graph shows the results of 1000 model runs as a heat map, the right graph shows the resulting coral cover estimates in 2100 as a frequency distribution, and the red dashed horizontal lines are 95% credible intervals on the model output distributions.

**Figure 6:**
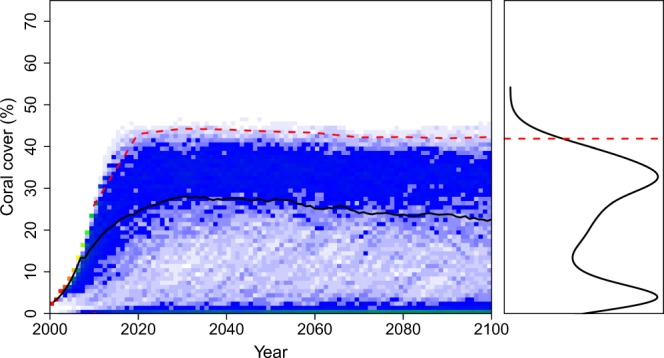
Predictions of % coral cover in Sesoko Island, southern Japan, with *high lambda* = *0*.*039* (Equation 2) for sea-surface temperatures predicted under climate-change scenario RCP 8.5 Wm^−2^ (i.e., 3.7 °C), and *high gamma* = *0*.*005* (Equation 1a*) and high epsilon* = 8 (i.e., with high intensity of thermal anomalies). The left graph shows the results of 1000 model runs as a heat map, the right graph shows the resulting percentage coral cover estimates in 2100 as a frequency distribution, and the red dashed horizontal lines are 95% credible intervals on the model output distributions.

**Figure 7:**
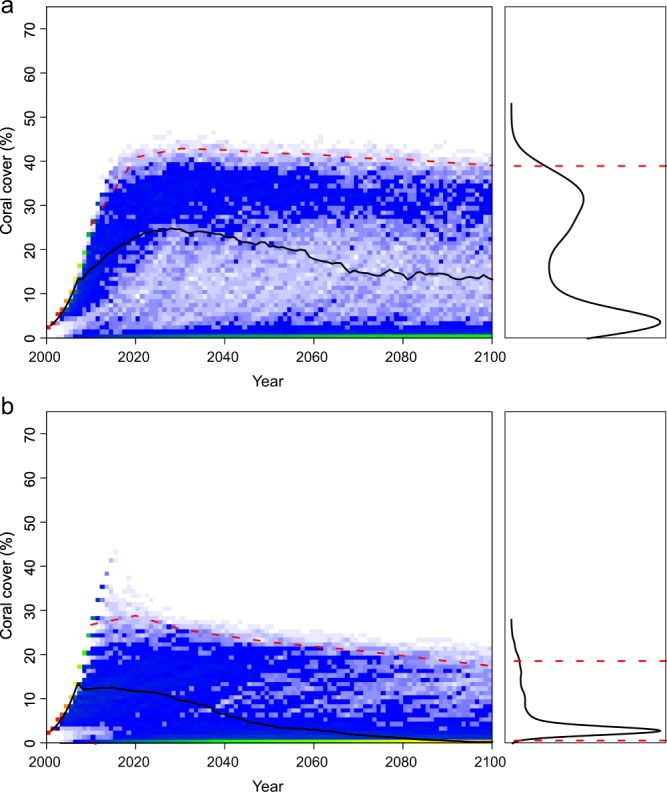
Predictions of % coral cover in (**a**) Sesoko Island, southern Japan and (**b**) Scott Reef, Western Australia under climate-change scenario RCP 8.5 Wm^−2^ (i.e., 3.7 °C) with thermal stress return periods input under *high frequency* (6 years) *with high lambda* = 0.039, *high gamma* = 0.005, and *high epsilon* = 8. The left graph shows the results of 1000 model runs as a heat map, the right graph shows the resulting percentage coral cover estimates in 2100 as a frequency distribution, and the red dashed horizontal lines are 95% credible intervals on the model output distributions.

Considering a negative effect of high sea-surface temperature on coral populations, the λ-values (Equation 2), or the ‘climate-change coefficients’, were estimated at 0.015, 0.021, and 0.039 (Table 1), for RCP 4.5, RCP 6.0, and RCP 8.5, respectively. Increases to the thermal susceptibility, *γ*, in Equation 1a, resulted in reductions in overall coral cover (compare Fig 3a to 5). Similarly, increasing the ‘extreme-event coefficient’, resulted in bimodal responses, with some model runs predicting population collapse, whereas other runs predicted recovery (Fig. 6). When the frequency of thermal-stress events was increased from 3–9 years to 3–6 years, the corals did not recover, particularly when thermal anomalies also increased in intensity (Fig. 7a), as is expected under a business-as-usual climate-change scenario (RCP 8.5). It is notable that when the two localities, with different coral recovery rates, were modeled, they showed different responses; Western Australia, with lower coral recovery rates (*r* = 0.17), completely collapsed under high frequency and intensity temperature anomalies (Fig. 7b), whereas reefs in southern Japan, with considerably higher coral recovery rates (*r* = 0.39), maintained low coral cover (Fig. 7a).

## Discussion

Forecasting when and where thermal stresses will occur is particularly challenging because of stochastic fluctuations in short-term inter-annual climatic cycles, and because of regional differences in both the ocean temperature and the rates of change in ocean temperature caused by global warming^38,39^. Still, while such short-term climate events are unpredictable in any given year, they are predictable over the course of a decade or more. Our model captured that predictability by using Poisson distributions that randomly selected years with anomalous thermal stress. Although the actual years when thermal-anomaly events will take place in the coming century are unknown, the intensity and frequency of the events used in our models are realistic enough to predict the responses of the coral populations through time.

Our nonlinear hybrid-stochastic-dynamical system allowed us to predict and compare a suite of coral-population responses under various climate-change scenarios. The resultant model predicted that coral populations responded more strongly to intensifying stochastic temperature anomalies than gradual increases in background global temperatures. Three parameters consistently affected the coral-population estimates in our model: (1) *ε*, which is the ‘extreme-event coefficient’, or the magnitude of the temperature anomaly; (2) *r*, which determines the rate of recovery of each coral population; and (3) γ, which is the impact rate of background temperature on the coral population.

Firstly, the coral-population models were particularly sensitive to the intensity of temperature anomalies (*ε*). Coral bleaching occurs during the most severe thermal-stress events^10^ when summer temperatures are exacerbated by climatic phenomenon, particularly El Niño events; although recently, thermal-stress events have been also occurring during non-El Niño years^5^. Recovery rates dampened through time when the intensity (*ε*) and the frequency of thermal-stress events increased, which are predicted to occur under the business-as-usual climate-change scenario RCP 8.5^38^. Our results showed that after intense and frequent thermal-stress events, the coral populations often hovered around <10% coral cover, from which the populations were unlikely to recover. Extremely low coral cover may reduce larval supply, which would further reduce recruitment through the Allee effect, with fitness declining in step with declines in population size.

Secondly, the data used to parameterize the model showed that coral populations in the two locations recovered at different rates. Coral recovery is dependent on numerous conditions, including recent history and what remains on the reef, and the period of recovery between thermal-stress events^25^. Indeed, the capacity of a population to regrow from remnant fragments has a considerable impact on the recovery rates of coral populations^40^. For example, small isolated remnants of massive *Porites* species regrow rapidly after disturbances^41^, but isolated remnants of corymbose *Acropora* species rarely regrow^27^. Consequently, corymbose *Acropora* populations are mostly dependent on larval recruitment for population recovery, whereas massive *Porites* can regrow from local remnants. These differences were reflected in the high variance in the *Acropora* response, compared with the lower variance of *Porites* response (Fig. 4). Recovery on a particular reef also depends upon the location of the nearest larval source. Scott Reef is considerably more isolated than Sesoko Island, which is adjacent to Okinawa where remnant coral populations survived through the 1998-thermal stress event. Isolation may have played a role in the differences in recovery rates between Scott Reef and Sesoko Island.

Thirdly, our model showed that populations with high γ values were more sensitive to high temperatures than populations with low γ values (Fig. 5). These results are not surprising since reef ecologists have long known that coral species differ in their tolerance to temperature anomalies^11^. Still, γ-values were strongly coupled with *r*-values — the two are not independent of each other. In fact, if we assume that *T* is constant, Equation 1a has two equilibrium points, at P_1_ = 0 and at P_2_=$\,\frac{K}{r}\,(r-\gamma T).$ On the one hand, P_2_ must be positive for the population to survive, but when $\frac{\gamma T}{r}=1$, then P_2_=0, which effectively becomes P_1_, and is therefore a bifurcation point. On the other hand if $\frac{\gamma T}{r} < 1$, then P_2_ > 0, the population is positive and at a stable equilibrium. Therefore, the long-term response of a population to temperature is dependent on the dynamics between the population’s sensitivity to temperature and its capacity to recover.

The model also showed considerable fluctuations in *Acropora* populations compared with the more stable *Porites* populations. Further studies on thermal tolerance may lead to more realistic and dynamic distributions of thermal tolerance γ values, which may even vary geographically. For example, modeling coral populations on isolated reefs with low genetic diversity^42^ will most likely predict leptokurtic distributions for γ values, with little variance, which may project considerably worse outcomes for those populations than for populations with high genotypic diversity.

Although carrying capacities, or equilibrium points, on coral reefs have rarely been quantified and discussed, coral-reef habitats do vary in their carrying capacity. For example, Gouezo *et al*., ref.^43^, recently showed long-term stability on the reefs of Palau, with the greatest differences in coral cover being apparent among habitats. Over a 15-year period, the nearshore reefs of Palau at 3 m depth supported ~50% coral cover, whereas the outer reefs supported on average 40% coral cover, and the patch reefs supported on average 20% coral cover^43^. In the present study, the carrying capacities (*K*) were similar, but were higher in Western Australia (64%) than in southern Japan (53%), although there was considerable variance around the means. This similarity should not be entirely surprising given the considerable overlap of the coral species composition, and the similarity of habitats between regions (van Woesik, pers. obs.). Yet, carrying capacities depend on numerous interacting processes, including exposure to waves and sedimentation rates. Although these processes have been rarely explored, we need further studies that more accurately quantify carrying capacities of coral reefs across habitats, and that determine the extent to which climate change is influencing those carrying capacities.

Notably, the model found characteristic bifurcations when temperature anomalies increased in both intensity and frequency. These bifurcations indicate that there is some uncertainty in reef responses to thermal-stress events, which may lead to either reef recovery or collapse. That collapse may be, in turn, dependent on specific thresholds, beyond which recovery is unlikely. The thresholds and bifurcations may also offer an estimate of just how little atmospheric CO_2_ it takes before ocean temperatures drastically increase a coral population’s probability of collapsing.

Thermal anomalies are becoming increasingly common. Especially prominent were the recent back-to-back bleaching events on the northern and central Great Barrier Reef in 2016 and 2017^5^. Estimates of recovery rates on the Great Barrier Reef are still preliminary and mostly remain unknown, although new evidence suggests that recovery rates on the Great Barrier Reef are becoming suppressed with chronic disturbances^44^. Our study provides a guide as to what recovery rates may be expected under future emissions scenarios. A rate of less than r = 0.38, or its discrete-equation equivalent of lambda 1.46, may indicate that the system is deteriorating and that the corals have already passed a critical threshold and lost their capacity to recover^20^. Understanding rates of recovery has become the cornerstone of resilience studies^21,22^. On contemporary reefs, increases in ocean temperature, and potential changes in inter-annual temperature cycles, are spatially variable across ocean regions^45,46^. Our model shows that coral populations are most sensitive to both the intensity and frequency of thermal-stress events, rather than to incremental ocean warming. However, ocean warming is likely to increase both the intensity and frequency of thermal-stress events. These thermal anomalies also may become more frequent outside of El Niño events^5^, considerably shortening recovery periods. These temperature thresholds, and the recovery rates of coral populations, are solely dependent on the rates of future greenhouse-gas emissions. Therefore, to ensure the best coral - recovery rates, greenhouse-gas emission rates need to track RCP 6.0 or lower.

## Electronic supplementary material

Appendix 1. Algorithm.

Supplementary Dataset 1
