# Supplementary material for: Predicting coral dynamics through climate change
Source: Sci Rep. 2018 Dec 20;8:17997. doi: 10.1038/s41598-018-36169-7 (PMC6301976; doi:10.1038/s41598-018-36169-7)
Supplement: Supplementary file 1 — Appendix 1. Algorithm. [file 41598_2018_36169_MOESM1_ESM.pdf]

Predicting coral dynamics through climate change

Robert van Woesik, Semen Köksal, Arzu Ünal, Chris W. Cacciapaglia,
Carly J. Randall

Appendix 1 Algorithm for the nonlinear hybrid stochastic dynamical system model.

Define the temperature function as

$$T(t) = I_{ave}\left(a_1\, cos(t) + a_2 sin(t)\right) + \lambda t + a_3$$

Set the initial values for

t_0 ; K; r ; P_0

Start the following loop:

DO for $i = 1, \dots \dots, N$ $(N\ is\ the\ \#\ of\ years\ we\ want\ to\ run\ the\ simulation)$

Choose t_k $(the\ years\ of\ extreme\ events)$ from the Poisson Distribution with $\lambda = 3, 6\ and\ 9$ for all, moderate and strong/very strong anomalies, respectively.

Solve $\frac{dP}{dt} = r\,P\left(1 - \frac{P}{K}\right) - \gamma\, T(t)\, P$ for $t_0 \leq t \leq t_k$

Graph and Store the graph of $P(t)$ for $t_0 \leq t \leq t_k$

Choose ε as a random real number from the intervals $[\,0.5, 4], [1, 4]$ and $[1.5,4]$

Define $P(t) = P(t_k)e^{f(\varepsilon)(t - t_0)}$

Choose h from the Gamma Distribution with $[4.92, 1/115]$

Graph and store the graph of $P(t)$ for $t_k \leq t \leq t_k + h$

Set $P_0 = P(t_k + h)$

Choose r from the Beta Distribution with $\alpha = 9.96$ and $\beta = 13.47$

Set $t_0 = t_k + h$

$i = i + 1$

END DO
